# The naturally occurring radioactivity of ‘scalar energy’ pendants and concomitant radiation risk

**DOI:** 10.1371/journal.pone.0250528

**Published:** 2021-06-01

**Authors:** Halmat Jalal Hassan, Suhairul Hashim, Mohamad Syazwan Mohd Sanusi, Mohamad Hidayat Jamal, Sitti Asmah Hassan, David Andrew Bradley, Rafael García-Tenorio, Rozman Mohd Tahar

**Affiliations:** 1 Department of Physics, Faculty of Science, Universiti Teknologi Malaysia, UTM, Skudai, Johor, Malaysia; 2 Department of Physics, College of Education, University of Sulaimani, Sulaimani, Kurdistan, Iraq; 3 Ibnu Sina Institute for Scientific and Industrial Research (ISISIR), Universiti Teknologi Malaysia, UTM, Skudai, Johor, Malaysia; 4 Faculty of Engineering, Universiti Teknologi Malaysia, UTM, Skudai, Johor, Malaysia; 5 Centre for Applied Physics and Radiation Technologies, Sunway University, Bandar Sunway, Selangor, Malaysia; 6 Department of Physics, University of Surrey, Guilford, United Kingdom; 7 Department of Applied Physics II, ETSA, University of Seville, Seville, Spain; 8 Centro Nacional de Aceleradores, CNA, University of Seville-J. Andalucía-CSIC, Seville, Spain; 9 Atomic Energy Licensing Board, Dengkil, Selangor, Malaysia; Semmelweis University, HUNGARY

## Abstract

Forming part of a study of radiological risk arising from use of radioactive consumer products, investigation is made of pendants containing naturally occurring radioactive material. Based on use of gamma-ray spectrometry and Monte Carlo (MC) simulations, the study investigates commercially available ‘scalar energy pendants’. The doses from these have been simulated using MIRD5 mathematical phantoms, evaluation being made of dose conversion factors (DCFs) and organ dose. Metallic pendants code MP15 were found to contain the greatest activity, at 7043 ± 471 Bq from ^232^Th, while glass pendants code GP11 were presented the greatest ^238^U and ^40^K activity, at 1001 ± 172 and 687 ± 130 Bq respectively. MP15 pendants offered the greatest percentage concentrations of Th, Ce, U and Zr, with means of 25.6 ± 0.06, 5.6 ± 0.005, 1.03 ± 0.04 and 28.5 ± 0.08 respectively, giving rise to an effective dose of 2.8 mSv for a nominal wearing period of 2000 h. Accordingly, these products can give rise to annual doses in excess of the public limit of 1 mSv.

## Introduction

As with other life forms, humans are continually exposed to ionizing radiation from natural radionuclides, including from terrestrial media, building materials, water, air and food. The International Atomic Energy Agency (IAEA) defines naturally occurring radioactive material (NORM) as “Radioactive material containing no significant amounts of radionuclides other than naturally occurring radionuclides”; included in this are materials in which the activity concentrations of the naturally occurring radionuclides have been changed by a process [1].

Scalar energy products, a recent health fad introduced into local and online markets in Malaysia, the country in which present study has been carried out, are positioned as pendants that when worn offer an alternative form of medicine. At the outset, let it be noted that the term scalar energy finds no conventional scientific definition, considered to be meaningless jargon by the present authors. The manufacturers of the pendants state them to be composed of volcanic materials and/or other minerals, the purveyors and manufacturers linking the supposed health benefits with negative ion technology and quantum science, also using further jargon such as energy quantum pendants and volcanic lava energy. The claimed health benefits include improved circulation, stamina and flexibility, with an ability to detoxify and enhance energy levels, also being linked with prevention of cancer [2]. Additional claims include that they are a way to maintain health, balance energy and improve emotional well-being, also protecting against electromagnetic fields [3].

As previously shown, such products contain thorium and uranium, concentrated in activity [4]. Given that over time risks have been identified, NORM has become increasingly subject to monitoring and regulation [5]; the IAEA safety guide “Radiation Safety for Consumer Products” [6] recommending the use of such items to first be justified. The pendants are apparently devoid of any scientific evidence of benefit to the general public [6, 7]. In order to limit radiation exposure from NORM-added consumer products, the Malaysian Atomic Energy Licensing Board (AELB) instituted control via its technical document LEM/TEK/69 [8]. This was preceded by the United States Environmental Protection Agency (EPA) guidelines for regulation of NORM [9]. Despite such concerns, globally harmonized regulations have yet to be established, regulating and controlling radioactive content consumer products [10].

The purpose of present study has been to investigate the pendants, the activity of the long-lived radionuclides ^238^U, ^232^Th and ^40^K being measured and potential dose to wearers being evaluated via Monte Carlo (MC) simulation. In this regard, exposure was simulated via the use of MIRD5 mathematical male and female phantoms, with evaluation made of dose conversion factors (DCFs) and organ dose, focusing on assessing external exposure to wearers.

## Materials and methods

### Sampling and HPGe γ-spectrometry

**Fig 1:**
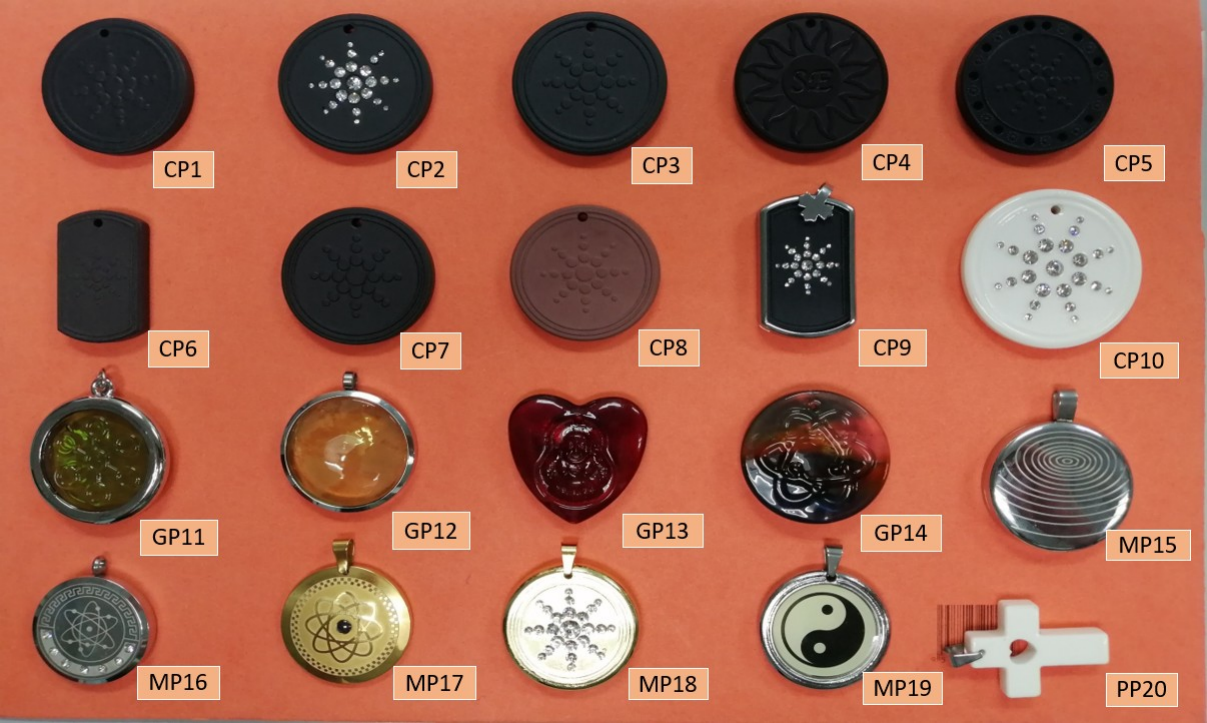
Scalar energy pendant samples.

The pendants are said to contain volcanic lava, zirconium, anions and monazite, monazite and zirconium being known to contain uranium and thorium [5]. For current study a total of 20 pendants were purchased online, upon delivery there being no evidence of information on the radioactivity contained within. The products were ten ceramic-based pendants (assigned codes CP1 to CP10), four glass-based pendants (codes GP11 to GP14), five metallic-based pendants (code MP15 to MP19), and one pendant with an outer plastic cover, code PP20 (Fig 1).

**Fig 2:**
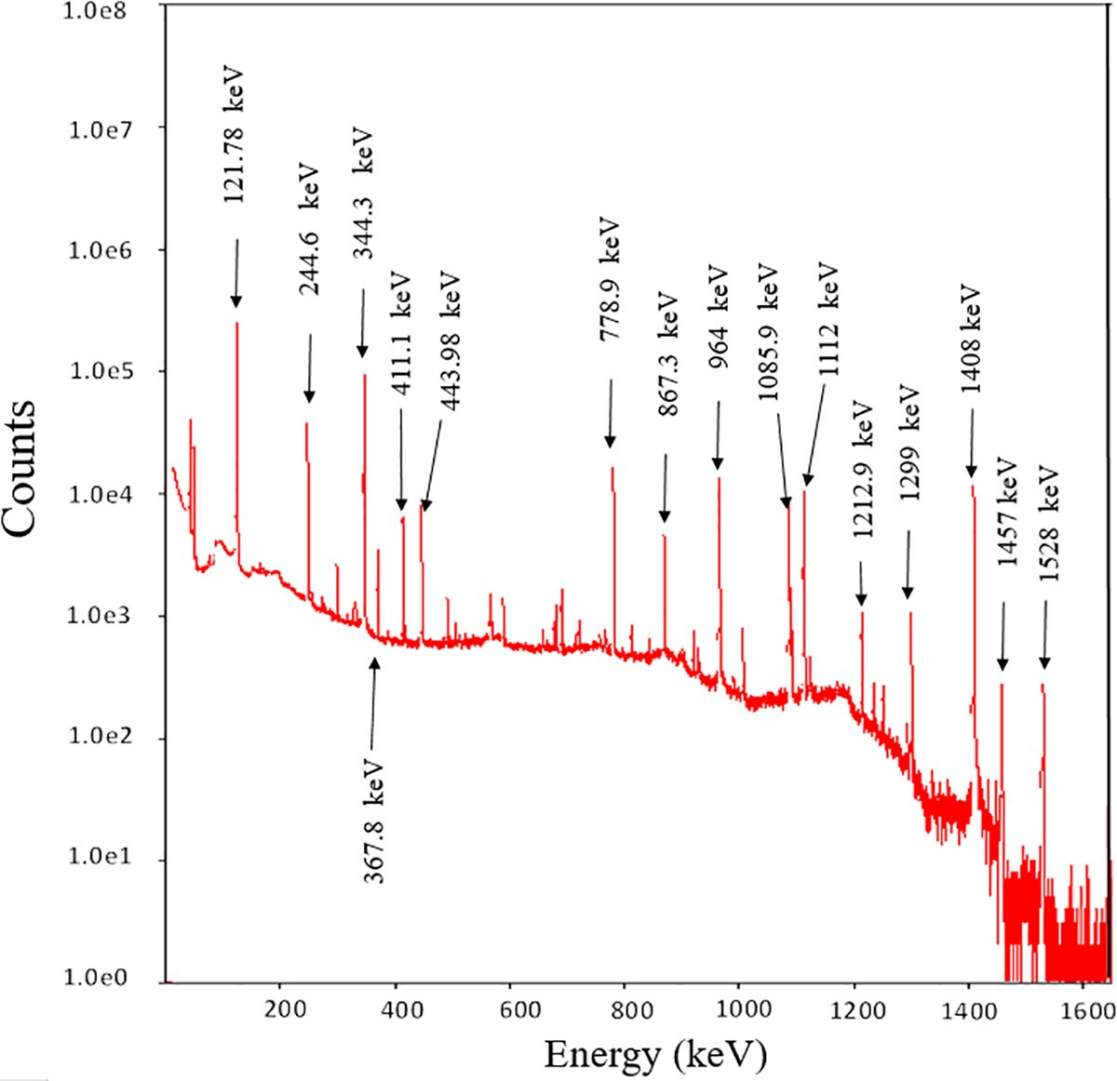
Calibration energy spectrum (standard point source ^152^Eu).

Using a well-shielded high purity Ge (HPGe) spectrometer, each pendant was counted for a period of 20 h, each firmly located within the shielding in a holder coaxially aligned with and 3 cm above the top cap of the HPGe detector, a geometrical configuration maintained for all pendants. The gamma emissions were directly measured using an ORTEC GEM Series P-type coaxial HPGe spectrometer (GEM20-76-LB-C-SMPCFG-SV-LB-76; 33% relative efficiency; 1.8 keV FWHM at 1332 keV), providing high-performance gamma spectroscopy within the photon energy range 40 keV to several MeV. The system, equipped with a Mobius cooling system, also uses high-resolution gamma spectroscopy software (VISION version 8) for spectral analysis. The spectra, collected over 16380 channels, were calibrated using a ^152^Eu standard point source, providing a wide range of photon energies (121.78, 244.6, 344.3, 411.1, 778.9, 867, 964, 1112 and 1528 keV) see (Fig 2).

**Fig 3:**
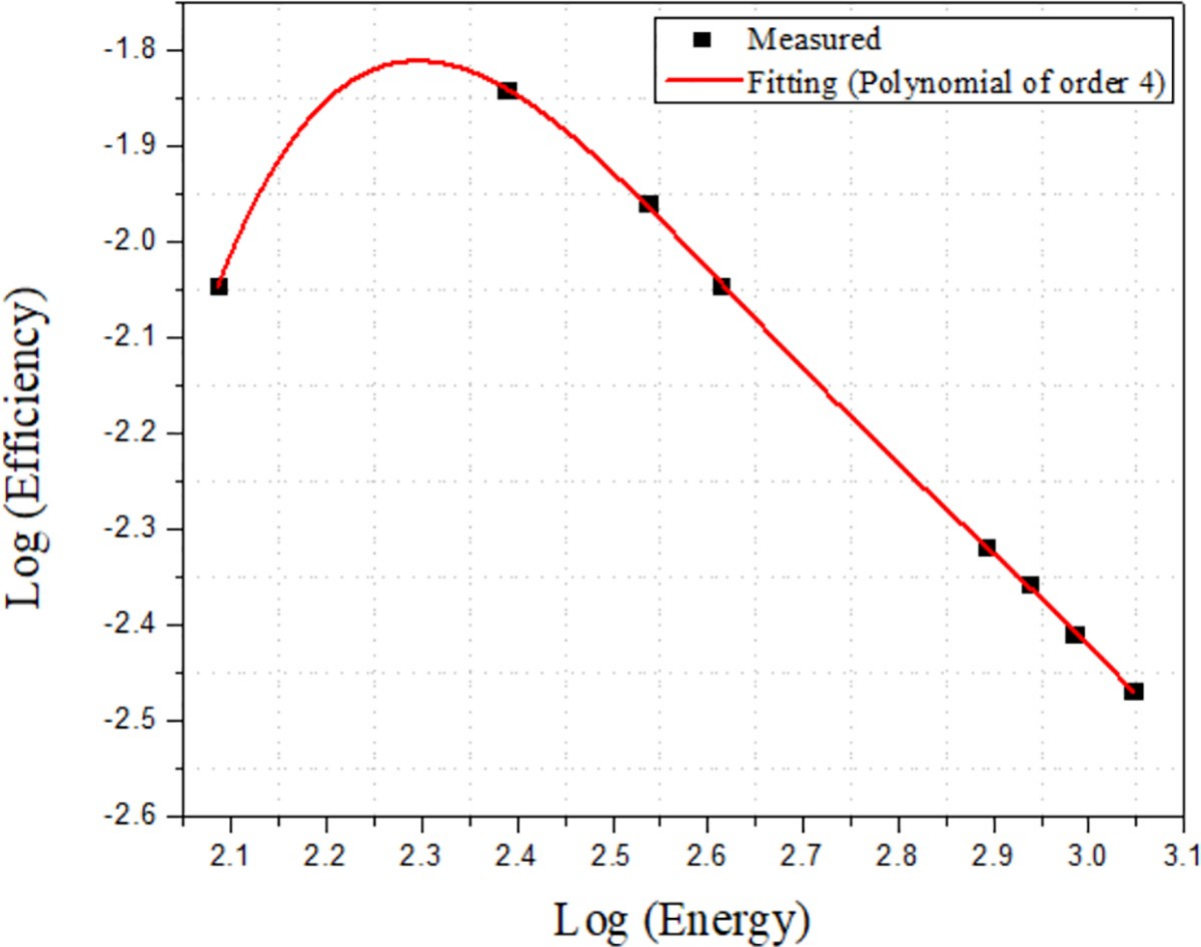
Energy efficiency of HPGe detector (standard point source ^152^Eu).

The counting efficiencies of the ^238^U series for these measurement conditions was 1.1% for ^214^Pb (351.8 keV) and 0.64% for ^214^Bi (609.3 keV), while for the ^232^Th series, they were 1.52% for ^212^Pb (238.4 keV), 1.24% for ^228^Ac (338.2 keV), 0.6% for ^212^Bi (727 keV), 0.71% for (583 keV), 0.16% for ^208^Tl (2614 keV), and 0.2% for ^40^K (1460 keV). With the exception of ^40^K, all gamma lines have a high branching ratio [11]. The efficiency curve (Fig 3) covers the photon energy range 121.78–1528 keV with a fitting polynomial of order 4, as given by Alnour et al. [12] and Gouda et al. [13], shown in (1) below:

$$log\;(\varepsilon )=\sum_{i=0}^{4}\;c_{i}.log\;{\left(E\right)}^{i}$$ (1)

with (*ε*) the full energy peak efficiency at energy E and *c_i_* the fitting coefficients determined via calculations. Interpolation was used to calculate the absolute efficiency of unknown radionuclides in the samples.

### Elemental characterization of pendants

For elemental analysis, the pendants were analysed using a Cartesian Geometry Energy Dispersive X-Ray Fluorescence (ED-XRF) spectrometer (model; NEX CG–CG1240), XRF offering non-destructive investigation in determining elemental content, including of consumer and food products [14–16]. The samples were prepared in homogenized powder form. XRF analysis of four such pendants has previously been carried out by Pabroa et al. [2].

### Monte Carlo (MC) simulation and evaluation of Annual Effective Dose (AED)

**Fig 4:**
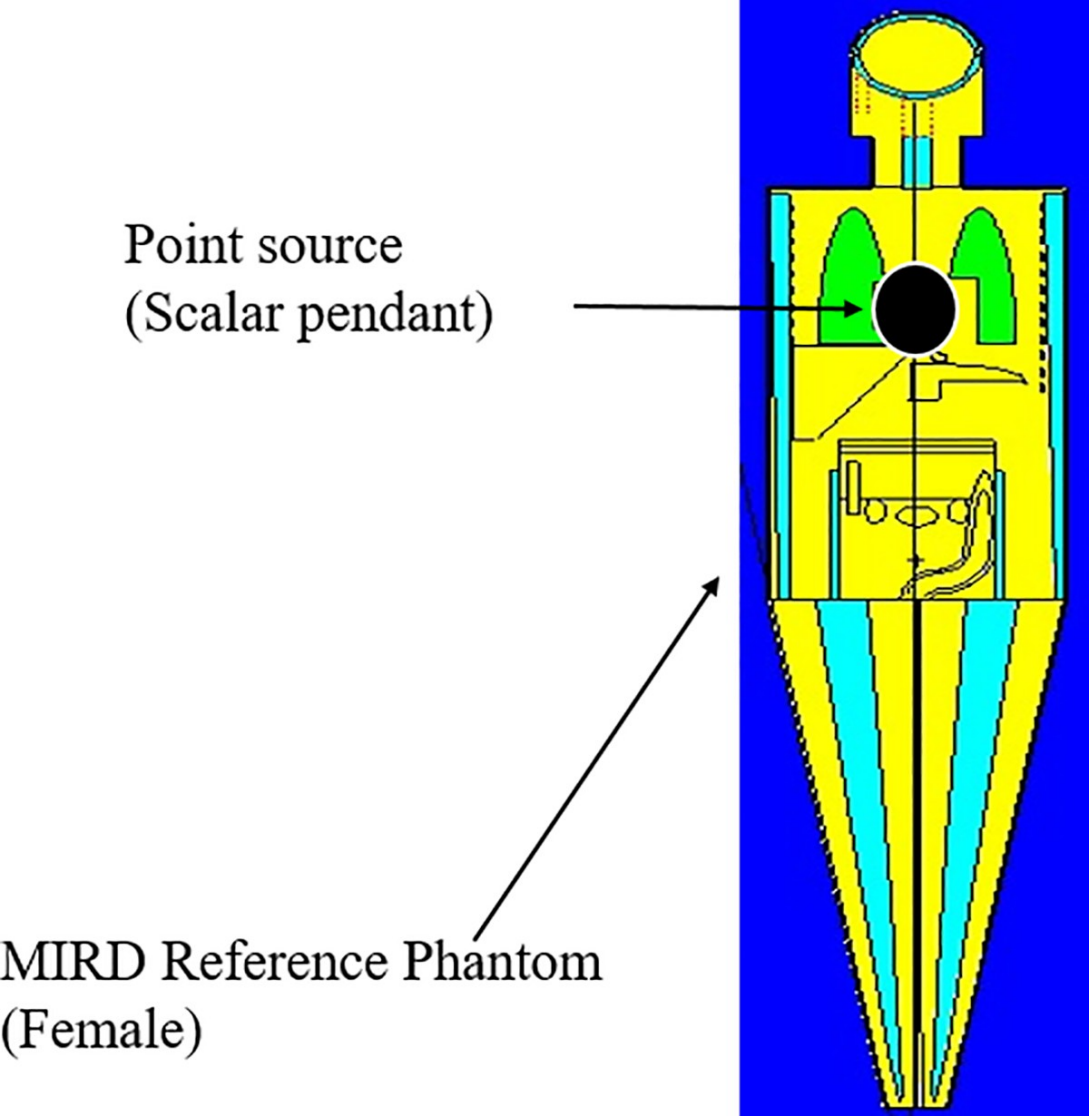
MIRD human phantoms for a scalar pendant, as used in MC simulation.

To estimate the equivalent organ doses and annual effective dose (AED) arising from use of the pendants simulation was undertaken using the Monte Carlo N-Particle radiation transport code version MCNP5 (Los Alamos National Laboratory), also involving use of Medical Internal Radiation Dose (MIRD) mathematical phantoms, male and female, with the female version shown in Fig 4 [5, 17, 18]. The male phantom is 178 cm tall, weight 91 kg, while the female phantom is of height 168 cm and weight 72 kg. The pendant was taken to be a sphere of 2 cm diameter, dose evaluations being carried out with the pendant located on the chest. Where, color of the phantom represents different composition and density of tissues and bone; green, yellow and aqua represent lung tissue, soft tissue, and bone, respectively.

### Dose calculation

#### Estimation of equivalent organ dose and effective dose, using (DCFs)

The radiological protection quantity equivalent dose denoted *H_T_*, defined by:

$$H_{T}=\Sigma \;w_{R}\;D_{T,R}$$ (2)

with *H_T_* (Sv) the equivalent dose of organ or tissue T, *w_R_* denoting the radiation weighting factor, for gamma radiation is unity (1), and *D_T,R_* (Gy) the mean absorbed dose in an organ or tissue T, due to gamma radiation [19], and therefore the effective dose, E (Sv), defined by a sum of weighted tissue equivalent dose as:

$$E=\Sigma \;w_{T}\;H_{T}$$ (3)

Where *w_T_* is the tissue weighting factor for tissue T, and summation *w_T_* is unity (1) [19–21].

The new Dose Conversion Factors (DCFs), calculated from the Monte Carlo simulation for a wearer of pendant. Where the (DCFs) formed the coefficient,*F_T_*, the equivalent dose in organ or tissue per unit integrated exposure. From this coefficient *F_T_*, the equivalent dose *H_T_* for any organ or tissue can be calculated as:

$$H_{T}=A\times t\times F_{T}$$ (4)

Where *A* (Bq/pendant) represents the radioactivity from the pendant, *t* represents the exposure time (hours), (8 hr/day * 5 days/week * 50 weeks/year) [5], and coefficient *F_T_* (Sv h^-1^ per Bq/pendant) represents the equivalent dose in organ or tissue per unit integrated exposure [3].

#### Estimation dose rate using HPC software

The dose-rate in air, bone, and muscle, calculated for the wearer, was obtained using Syberad’s Health Physicist’s Companion program. The program executable file is HPC.exe. Syberad’s reference number is HP-CD1. This database is a user-friendly software program designed for radiation protection professionals, useful in obtaining information in respect of more than 2800 nuclides [22]. In particular, it can be used to calculate the dose-rate for any radionuclide, for a given specific activity, at any distance, with various shielding provision [3]. Dose-rates were calculated for the radionuclides ^238^U, ^232^Th and ^40^K in the pendant, at close-up distance from the user.

#### Measuring dose equivalent using a survey meter

The dose equivalent from the pendants was also measured, use being made of a calibrated identiFinder 2, FLIR Survey Meter, recording exposure dose-rates (in μSv/h), also identifying the radionuclides contained in the pendants.

## Results and discussion

### Measurements of the ^238^U, ^232^Th and ^40^K activity in the pendants

**Fig 5:**
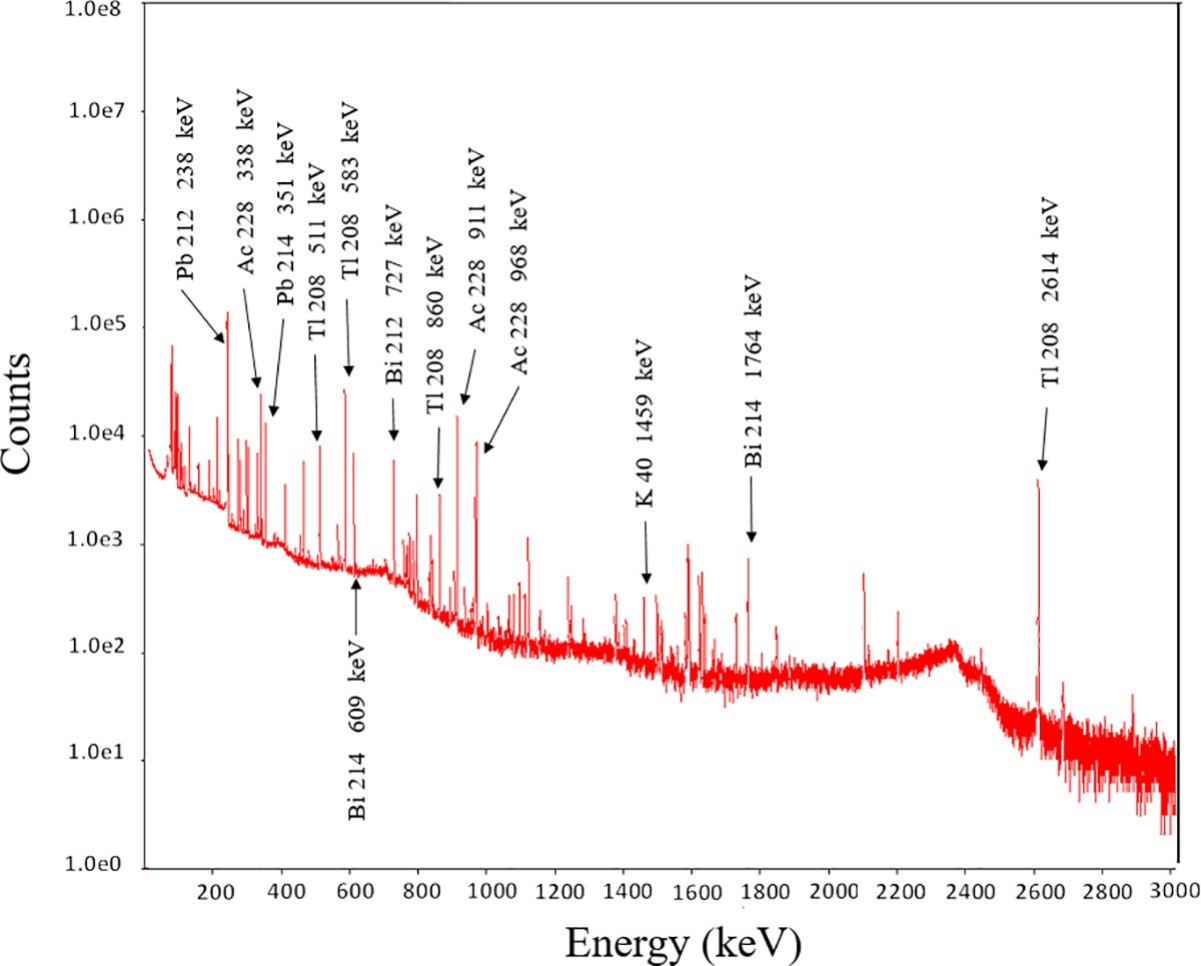
Example spectra obtained from an MP15 metallic scalar pendant sample.

Using the ORTEC GEM Series P-type coaxial HPGe detector, gamma spectrometry measurements were made for each of the 20 pendants, with an example shown in Fig 5 for pendant MP15, identifying ^40^K activity as well as U and Th series nuclides, including ^214^Pb, ^214^Bi, ^212^Pb, ^228^Ac, ^208^Tl (Table 1). The results for ^238^U were estimated from the average of results for gamma emissions from ^214^Pb (295 keV and 351.8 keV) and ^214^Bi (609 keV and 1764 keV). The results for ^232^Th were estimated from the average of the gamma emissions of ^212^Pb (238 keV), ^228^Ac (338 keV and 911 keV), ^212^Bi (727 keV), and ^208^Tl (583 keV and 2614 keV), secular equilibrium being assumed [23].

**Table 1:** Activity (in Bq/pendant) of U-238, Th-232 and K-40 in the pendants.

Sample	U-238	Th-232	K-40
Ceramic Pendants			
CP1	13.4 ± 2.3	148 ± 10	155 ± 27
CP2	459 ± 77	885 ± 59	308 ± 56
CP3	75 ± 12	652 ± 66	278 ± 50
CP4	138 ± 23	992 ± 104	313 ± 56
CP5	185 ± 32	1515 ± 169	293 ± 53
CP6	106 ± 18	748 ± 147	104 ± 19
CP7	172 ± 29	1158 ± 122	295 ± 53
CP8	373 ± 63	2785 ± 180	260 ± 50
CP9	53 ± 9	356 ± 23	97 ± 18
CP10	55 ± 10	265 ± 18	116 ± 21
Glass Pendants			
GP11	1001 ± 172	6969 ± 483	687 ± 130
GP12	447 ± 76	2805 ± 185	262 ± 48
GP13	288 ± 48	1906 ± 124	164 ± 30
GP14	440 ± 75	2499 ± 166	262 ± 51
Metallic Pendants			
MP15	927 ± 157	7043 ± 471	536 ± 100
MP16	702 ± 120	5348 ± 363	514 ± 95
MP17	9 ± 1.5	73 ± 5	73 ± 11
MP18	6 ± 1	41 ± 3	20 ± 3.5
MP19	22 ± 3	167 ± 11	20 ± 4
Oute Plastic Pendant			
PP20	14 ± 3	3552 ± 239	395 ± 72

Pendant MP15 showed the greatest activity, at 7043 ± 471 Bq from ^232^Th, while glass pendant GP11 presented the greatest activity for ^238^U and ^40^K, at 1001 ± 172 and 687 ± 130 Bq respectively. Across the same category of pendant considerable difference in radioactivity content can be observed. For elemental content, four pendants were analysed via use of an XRF spectrometer (Table 2).

**Table 2:** Elemental composition of pendants.

Element	Samples (% Concentration)			
	CP6	CP8	GP11	MP15
Na	1.95 ± 0.2	ND*	6.10 ± 0.01	ND
Al	13.9 ± 0.02	76.5 ± 0.07	5.95 ± 0.02	8.15 ± 0.01
Si	63.0 ± 0.06	7.78 ± 0.01	62.3 ± 0.07	9.99 ± 0.01
K	0.75 ± 0.02	1.10 ± 0.03	2.30 ± 0.02	2.10 ± 0.02
Ti	0.36 ± 0.01	3.20 ± 0.01	0.70 ± 0.001	0.90 ± 0.01
Cr	0.55 ± 0.003	0.82 ± 0.03	0.30 ± 0.002	0.20 ± 0.02
Fe	8.32 ± 0.003	2.90 ± 0.03	1.20 ± 0.003	2.49 ± 0.03
Co	0.03 ± 0.002	0.03 ± 0.001	0.02 ± 0.001	ND
Sr	0.04 ± 0.001	0.03 ± 0.001	0.02 ± 0.001	ND
Zr	0.60 ± 0.003	0.56 ± 0.003	1.80 ± 0.06	28.5 ± 0.08
U	0.05 ± 0.001	0.05 ± 0.001	0.45 ± 0.001	1.03 ± 0.04
Ra	0.02 ± 0.001	0.02 ± 0.001	0.01 ± 0.001	0.04 ± 0.001
Pb	0.03 ± 0.001	0.02 ± 0.001	0.10 ± 0.001	0.03 ± 0.001
La	0.06 ± 0.001	0.03 ± 0.001	0.10 ± 0.001	2.53 ± 0.003
Ce	0.12 ± 0.006	0.13 ± 0.01	0.70 ± 0.009	5.60 ± 0.005
Th	0.89 ± 0.002	1.10 ± 0.06	12.6 ± 0.005	25.6 ± 0.06
Eu	0.24 ± 0.005	ND	ND	1.0 ± 0.005
Ba	0.02 ± 0.001	0.03 ± 0.001	0.10 ± 0.01	0.20 ± 0.001
Gd	0.07 ± 0.003	0.07 ± 0.001	0.70 ± 0.01	0.40 ± 0.002

ND*: Not Detected

Table 2 shows pendants CP6 and GP11 to be made primarily of Si and Al, while Al is the primary constituent of CP8 and for MP15 the primary component is Zr. NORM and rare earth elements (REEs) are found in all four analysed samples, MP15 recording the greatest concentration percentage of Th, Ce, U and Zr, at 25.6 ± 0.06, 5.6 ± 0.005, 1.03 ± 0.04 and 28.5 ± 0.08, respectively. The concentration (and activity) of U and Th for pendant MP15 were 10,090 ppm (872 Bq) and 260,000 ppm (7389 Bq) respectively. The results for samples MP15, as shown in (Tables 1 and 2) are comparable.

### Organ equivalent dose

**Fig 6:**
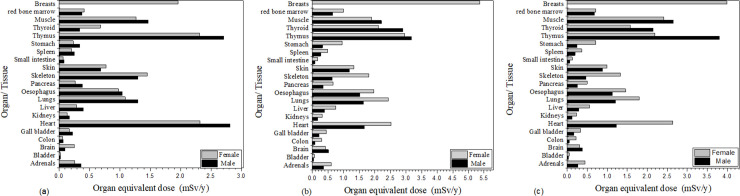
Organ equivalent dose (mSv/y) for 21 organs using the MIRD5 mathematical male and female phantoms, for: (a) CP8; (b) GP11; (c) MP15.

For pendants CP8, GP11 and MP15, Fig 6 shows results for 21 organs, the organ equivalent dose being calculated using the MIRD5 mathematical male and female phantoms. The thymus, thyroid, oesophagus, lung and heart are among the organs most greatly exposed, a result of their close distance to the source. For CP8, the highest organ equivalent dose was shown to be the heart, at 2.81 and 2.31 mSv y^-1^ for the male and female phantom respectively, assuming a nominal wearing period of 2000 hours in one year. The equivalent dose to the breast is also greater than that of other organs due to the area of exposure and close distance from the pendant, the pendant being simulated to be on the chest. In the case of GP11, the greatest organ equivalent dose was for the female breast, at 5.32 mSv y^-1^. For MP15 the muscle equivalent dose is also relatively high, at 2.65 and 2.45 mSv y^-1^ for the male and female respectively, due to the area exposed. Comparison of MC simulations were made with the only other known work, that of Lee et al. [5], using the ICRP reference phantom and MCNPX.

Table 3 shows equivalent doses (in mS/y), using Syberad’s Health Physicist’s Companion program, MP15 recording the highest annual doses, at 3.99, 4.08 and 4.24 mSv/y in air, bone and muscle, respectively. The HPC program calculated dose based on the wearer being exposed for a nominal period of 2000 hours per year. For CP8, the equivalent dose in muscle is 1.47 and 1.27 mSv/y for males and females respectively, comparable with the HPC result of 1.85 mSv/y (Table 3), in support of results obtained using the HPC program.

**Table 3:** Annual dose (in mSv/y) due to U-238, Th-232 and K-40, obtained using the HPC program.

Sample	Air	Bone	Muscle
Ceramic Pendant			
CP6	0.88	0.93	0.98
CP8	1.64	1.71	1.85
Glass Pendant			
GP11	3.59	3.74	4.02
Metallic Pendant			
MP15	3.99	4.08	4.24

### Evaluation of annual effective dose

**Fig 7:**
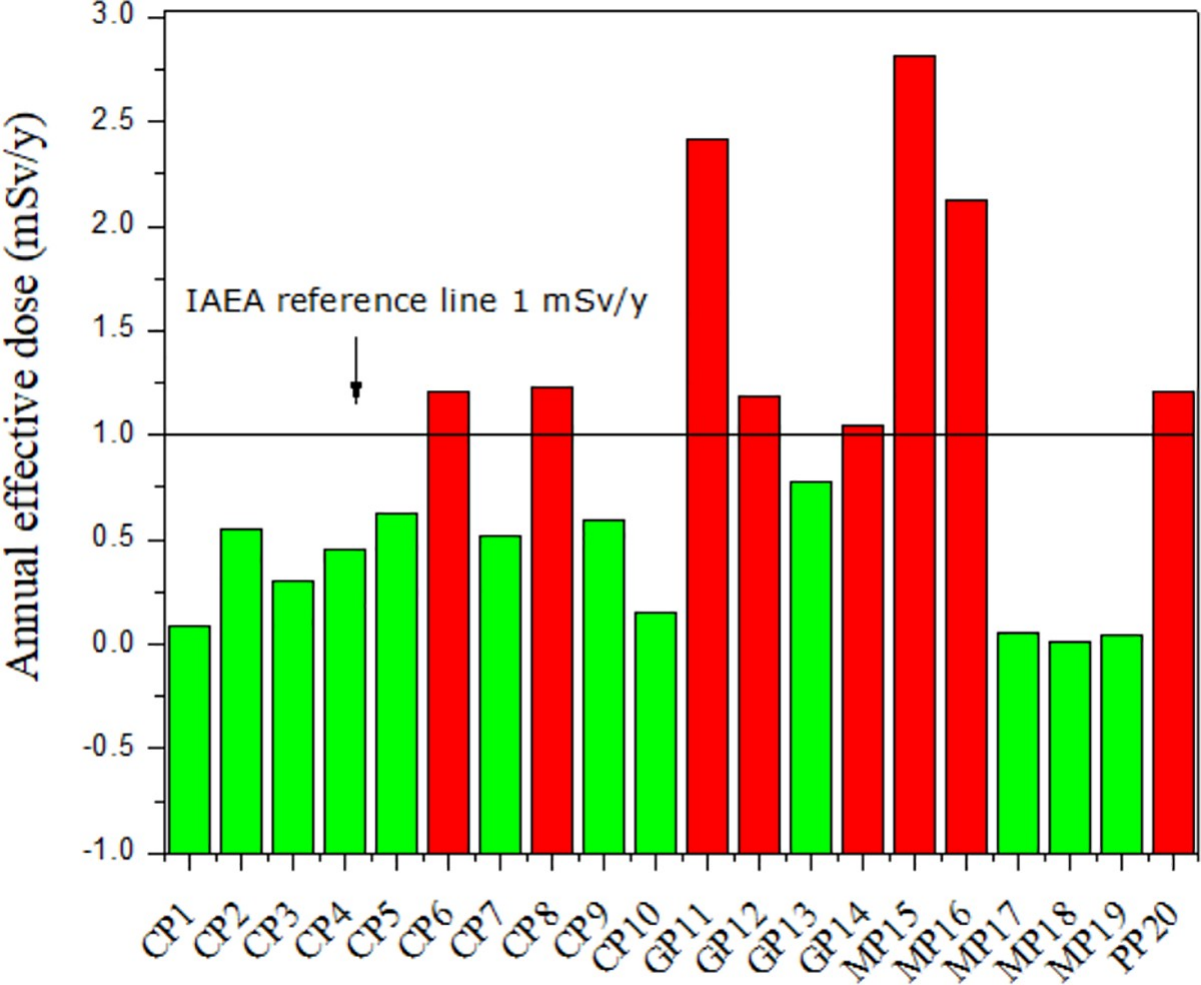
Annual effective dose for the 20 scalar pendants demonstrating measurable gamma activity.

The annual effective dose for the 20 pendants are presented in Fig 7, MP18 recording the lowest annual effective dose, at 10.6 μSv y^-1^, while pendant MP15 indicated the highest annual effective dose, at 2.81 mSv y^-1^. As in Fig 7, close contact with the pendants can infer annual effective doses in excess of the annual dose limit of 1 mSv/y for members of the public [6, 7]. The alpha emissions are essentially absorbed within the pendant, posing no threat to health, while beta emissions from the pendants will be mostly absorbed within the epidermal layers of the skin. Conversely, gamma radiation is deeply penetrating, involving large areas of tissue and organs.

The dose equivalent was also measured for the eight pendants capable of giving rise to annual effective doses in excess of 1 mSv/y. For this, use was made of a calibrated IdentiFinder 2, FLIR Survey Meter, recording dose rates in μSv/h and identifying the radionuclides contained within the pendants (Table 4). The highest dose equivalent, at 0.74 ± 0.1 μSv/h, was from MP15.

**Table 4:** Dose-rates from pendants, obtained using the IdentiFinder 2, portable meter.

No.	Sample	Dose rate (μSv/h)
1	CP6	0.25 ± 0.03
2	CP8	0.36 ± 0.07
3	GP11	0.52 ± 0.09
4	GP12	0.38 ± 0.05
5	GP14	0.3 ± 0.02
6	MP15	0.74 ± 0.1
7	MP16	0.41 ± 0.03
8	PP20	0.35 ± 0.04

Regarding the wearing period, we suggest 8 hours per day, 2000 hours per year, to be likely for those who purchase such items. For this, the annual dose reaches a value of 1.5 mSv y^-1^ for sample MP15, a value comparable with the MC results. In many countries, the use of radioactive materials in consumer products is regulated. As an instance a Council Directive of EURATOM in (Article 21), prohibits the sale of foodstuffs, toys, personal jewellery and cosmetics within which radioactive materials have been added. Unjustified exposures, as reported in ICRP-103, can be applied to a range of NORM consumer products. If the benefits of use of a consumer product to which NORM has been added cannot be shown to exceed the risk, then a ban would seem to be required [24, 25], as would certainly seem to be the case for the presently investigated products. Accordingly, a strong recommendation is made for prohibiting the pendant products in Malaysia.

## Conclusions

Using an HPGe detector, the radioactivity of 20 ‘scalar energy’ pendants were investigated. Pendants MP15 and GP11 recorded the greatest level of radioactivity, Th, U and K being found to be greatest in sample MP15. Estimation of the organ dose equivalent and annual effective dose was obtained using Monte Carlo simulation, leading to the conclusion that close contact with these pendants can infer annual effective doses of up to 3 mSv per year in excess of the dose constraint of 1 mSv y^-1^ for members of the public. While an absence of any justification for the use of these pendants is apparent, nevertheless these products are available for purchase in Malaysia, and that too in the absence of any indication of the items containing radioactivity. We strongly recommend that consideration be given to the prohibition of the importation and sale of these products.

## Supporting information

S1 Fig Detection of radionuclides using a Survey Meter, type identiFinder 2. (PDF)

S1 Table S1A. Elemental composition of energy pendants. S1B. Dose rate (μSv/h) of radioactivity in the energy pendant, using Survey Meter (IdentiFinder 2) portable detector.
(XLSX)

S1 File (ZIP)
