# Supplementary figures and images for: The naturally occurring radioactivity of ‘scalar energy’ pendants and concomitant radiation risk
Source: PLoS One. 2021 Jun 1;16(6):e0250528. doi: 10.1371/journal.pone.0250528 (PMC8168844; doi:10.1371/journal.pone.0250528)

**S1 Fig.** Detection of radionuclides using a Survey Meter, type identiFINDER 2.

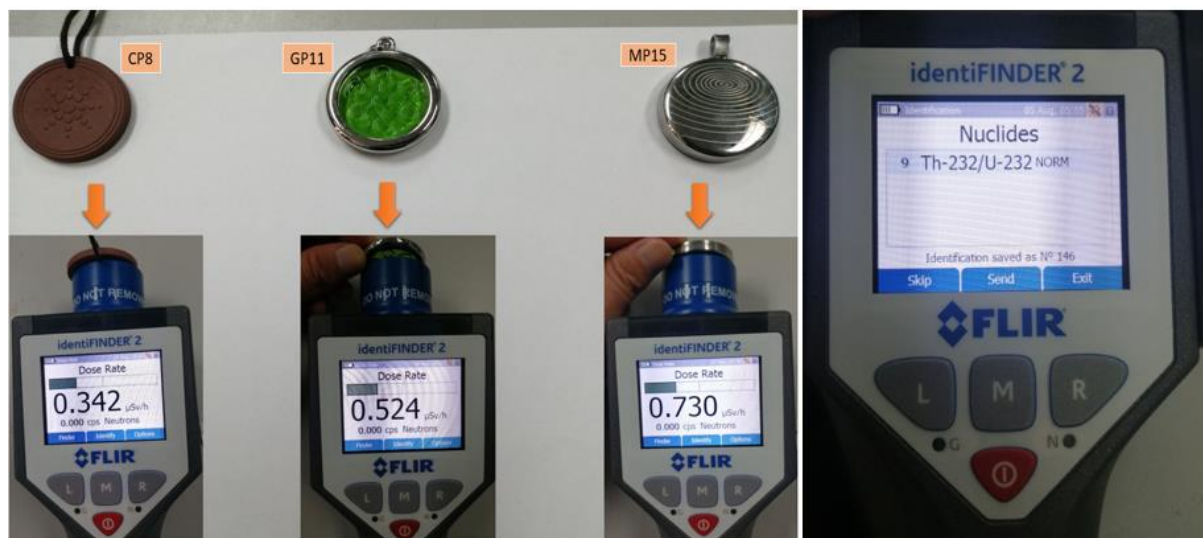

Supplement: S1 Fig — (PDF) [file pone.0250528.s001.pdf]
